# Supplementary material for: Improved assessments of bulk milk microbiota composition via sample preparation and DNA extraction methods
Source: PLoS One. 2022 Sep 15;17(9):e0267992. doi: 10.1371/journal.pone.0267992 (PMC9477292; doi:10.1371/journal.pone.0267992)
Supplement: Attachment — Submitted filename: PlosOne Response to Reviewers 8-13-22.docx [file pone.0267992.s001.docx]

Journal Requirements:

When submitting your revision, we need you to address these additional requirements.

1. Please ensure that your manuscript meets PLOS ONE's style requirements, including those for file naming. The PLOS ONE style templates can be found at

<https://journals.plos.org/plosone/s/file?id=wjVg/PLOSOne_formatting_sample_main_body.pdf> and

<https://journals.plos.org/plosone/s/file?id=ba62/PLOSOne_formatting_sample_title_authors_affiliations.pdf>

**Response:** We revised the paper to meet expectations for the PlosOne formatting style

2. Thank you for stating the following financial disclosure:

“The funders had no role in study design, data collection and analysis, decision to publish, or preparation of the manuscript.”

At this time, please address the following queries:

a)        Please clarify the sources of funding (financial or material support) for your study. List the grants or organizations that supported your study, including funding received from your institution.

b)        State what role the funders took in the study. If the funders had no role in your study, please state: “The funders had no role in study design, data collection and analysis, decision to publish, or preparation of the manuscript.”

c)        If any authors received a salary from any of your funders, please state which authors and which funders.

d)        If you did not receive any funding for this study, please state: “The authors received no specific funding for this work.”

Please include your amended statements within your cover letter; we will change the online submission form on your behalf.

**Response:** The Acknowledgements section was revised to include this information.

3. Thank you for stating the following in the Acknowledgments Section of your manuscript:

“The funding agency did not participate in study design, data collection, or interpretation of the data.” We note that you have provided additional information within the Acknowledgements Section that is not currently declared in your Funding Statement. Please note that funding information should not appear in the Acknowledgments section or other areas of your manuscript. We will only publish funding information present in the Funding Statement section of the online submission form. Please remove any funding-related text from the manuscript and let us know how you would like to update your Funding Statement. Currently, your Funding Statement reads as follows:

“The funders had no role in study design, data collection and analysis, decision to publish, or preparation of the manuscript.”

Please include your amended statements within your cover letter; we will change the online submission form on your behalf.

**Response:** We revised the Acknowledgements section and provide the funder information in the cover letter.

4. Please review your reference list to ensure that it is complete and correct. If you have cited papers that have been retracted, please include the rationale for doing so in the manuscript text, or remove these references and replace them with relevant current references. Any changes to the reference list should be mentioned in the rebuttal letter that accompanies your revised manuscript. If you need to cite a retracted article, indicate the article’s retracted status in the References list and also include a citation and full reference for the retraction notice.

**Response:** We revised the reference list to include additional citations relevant to the reviewer’s comments.

Additional Editor Comments:
Improved assessments of milk microbiota composition via sample preparation and DNA extraction methods
Overall the article is well written with clear presentation of results and discussion. As outlined by the reviewers expansion of details around sample type, revision of fig.1 and including information on the potential relevance of this study relative to other published literature will help strengthen the submission.

**Response:** We thank the editor for the support of our paper. We have revised the manuscript to address the concerns raised by the reviewers.

**Reviewers' comments:
Reviewer's Responses to Questions**

**Comments to the Author**

1. Is the manuscript technically sound, and do the data support the conclusions?
The manuscript must describe a technically sound piece of scientific research with data that supports the conclusions. Experiments must have been conducted rigorously, with appropriate controls, replication, and sample sizes. The conclusions must be drawn appropriately based on the data presented.

Reviewer #1: Yes

Reviewer #2: Yes

2. Has the statistical analysis been performed appropriately and rigorously?

Reviewer #1: Yes

Reviewer #2: Yes

3. Have the authors made all data underlying the findings in their manuscript fully available?
The [PLOS Data policy](http://www.plosone.org/static/policies.action#sharing) requires authors to make all data underlying the findings described in their manuscript fully available without restriction, with rare exception (please refer to the Data Availability Statement in the manuscript PDF file). The data should be provided as part of the manuscript or its supporting information, or deposited to a public repository. For example, in addition to summary statistics, the data points behind means, medians and variance measures should be available. If there are restrictions on publicly sharing data—e.g. participant privacy or use of data from a third party—those must be specified.

Reviewer #1: Yes

Reviewer #2: Yes

4. Is the manuscript presented in an intelligible fashion and written in standard English?
PLOS ONE does not copyedit accepted manuscripts, so the language in submitted articles must be clear, correct, and unambiguous. Any typographical or grammatical errors should be corrected at revision, so please note any specific errors here.

Reviewer #1: Yes

Reviewer #2: Yes

5. Review Comments to the Author
Please use the space provided to explain your answers to the questions above. You may also include additional comments for the author, including concerns about dual publication, research ethics, or publication ethics. (Please upload your review as an attachment if it exceeds 20,000 characters)

**Reviewer #1:** Improved assessments of milk microbiota composition via sample preparation and DNA extraction methods

General comments.
While there have been many studies reporting the composition of the “bovine milk microbiota” results have varied enormously partly due to a lack of standardized methods and this type of work is urgently needed. It is important to distinguish sample types – studies using samples collected from individual cow mammary glands need to be evaluated differently than samples collected from comingled, refrigerated bulk milk or cheese vats.

**Response:** We thank the reviewer for the encouraging works and recognition of our work.

Specific Comments
Title – This paper addresses results of studies that have evaluated unprocessed bulk milk microbiota (not individual quarter or composite milk samples from cows). While the concepts described in this paper likely apply to any bovine milk sample, it would help clarify the purpose of this paper to modify the title to read “Improved assessments of “unprocessed bovine bulk milk microbiota…”
**Response:** We revised the title to clarify that bulk milk was the focus of the work.

Introduction –
Line 50 –59: This paragraph refers to variation in sample type and should be expanded to focus on variation in the types of bovine milk that has been evaluated. The bovine milk microbiota has been evaluated on a variety of samples including bulk milk from single farms, comingled milk from processing vats, individual cow quarter milk samples, teat canal samples, and composite milk samples from cows. Variation in results is at least in part due to variation in the expected viable communities in these diverse samples. For example, it would be good to add a few comments about the challenges in aseptic collection of bovine milk samples (contamination from teat canal bacteria, sampling process and environment), and how various studies evaluating the bovine milk microbiota have used differing methods to collect milk samples. It is also important to note that the studies cited (references 16-18) all referred to raw BULK milk samples (not individual quarter milk samples from cows). Raw bulk milk contains bacteria that are acquired during the harvesting and storage process, thus results of studies describing the bovine milk microbiota that used bulk milk samples need to be differentiated from results of studies that use individual milk samples.

**Response:** We thank the reviewer for this insight and reminding us of this important point. We revised the Introduction and Discussion to explain the challenges associated with microbial analysis for different milk sample (collection) types.

Materials and Methods
L94 – why was 2% fat milk used? That is lower than that expected in unprocessed milk. Would you expect the lower fat concentration to influence your results?

**Response:** We used the UHT milk to minimize contamination of the BCMC and thus skewing of the data for the DNA extraction and purification tests.

L109 –“performed” not “preformed”

**Response:** Corrected.

L110 – delete “sample”
**Response:** Deleted

Results.
L212-213 – please add “good quality, raw “BULK” milk

**Response:**  “bulk’ has been added

L215-218 – this is very good information that will be useful to future researchers

**Response:**  Thank you!

Discussion - Please add a sentence or 2 to guide readers relative to applicablity of these results to other studies - especially those using quarter or composite milk samples collected directly from bovine mammary glands.

**Response:**  The Discussion has been revised to address the need to test the protocols for freshly expressed quarter/composite milk samples and several relevant recent publications on this topic have been included. We agree, also based on our own recent experience, that milk collected directly from bovine mammary glands has additional challenges for PCR amplification.

L304-308 – nice summary statement

**Response:**  Thank you!

Reviewer #2: I am writing to express my opinion regarding the study entitled “Improved assessment of milk microbiota composition via sample preparation and DNA extraction methods” from Marco and Xue.

In general, the article is well structured, with a very good explanation of the results and a well-defined discussion of the same. The study regards the assessment of a good standardized methodology for milk sampling collection in order to obtain high quality-data for microbiome composition studies. The authors tried to settle the best conditions for sampling, storing, and analyzing milk samples to determine a microbial composition without any biases due to the excess presence of certain bacteria compared to others.

In my opinion, the study deserves to be published, because it paves the basis of best practices when it comes to milk microbiome studies. There are just a few minor explanations and corrections that authors should make before the article can go for publication.

**Response:**  Thank you for the positive remarks and support of our paper.

Materials and Methods section
In this section, it is not clear why they prepared the mock community and then they inoculated the BCMC2 in milk samples. In general, the whole preparation of the study needs to be revised and clarified.

**Response:** We revised the materials and methods to explain the rationale to prepare the BCMC prior to inoculation into the milk. We also revised the order of the materials and methods section and tried to clarify further with revisions to Fig 1.

Paragraph 2.4: for example, to me, it was not clear why there was no milk in the sample storage condition comparisons, but just b authors mentioned the inoculation of the milk in the previous paragraph.

**Response:** Thank you for pointing this out. Because we collect the bacterial cells from milk samples prior to freezing, we did not include milk in the sample storage condition tests. However, we recognize that this may be regarded to be a limitation of the study and have revised the Discussion section to explain this constraint within the study design.

Fig. 1: this figure is intended to explain in an easier way the Materials and Methods of the study, but in my opinion, needs a little revision, to help the reader what has been done.

**Response:** Fig 1 was completely revised based on the suggestion and we believe it now more accurately shows the different experiments performed and described in the paper.

Line 159: Could you please explain why you used both qiime 1 and qiime 2? Did the results refer to which qiime? Moreover, you used the 2018.4 version of qiime 2, which is from 4 years ago. Could you try to explain why you choose to use an older version instead of a new one?

**Response:** QIIME 1.9.1 was only used to extract custom barcode sequences using the extract_barcodes.py script with no barcode error allowed. This was because at the time of the analysis, that function was not available in QIIME 2. The analysis parameters in the QIIME 2 2018.4 version was used based on a previous publication that compared a few different bioinformatics workflow for 16S rRNA gene bacterial community analysis (https://journals.asm.org/doi/full/10.1128/mSphere.00410-18). We expect the results and conclusions to stay the same even if a newer version of QIIME 2 is used.

Line 168: this is just a curiosity. Why did you use Greengenes instead of Silva of other databases?
**Response:** We used Greengenes because our prior publication showed it was more accurate than RDP (https://journals.asm.org/doi/full/10.1128/mSphere.00410-18). We now explain this in the Materials and Methods. We agree that Silva is a good and frequently used alternative, but for the purposes of continuity, we used Greengenes in this follow-up paper.

Results and Discussion sections
Those two sections are very well written and explained. Everything done for this study becomes very clear when I read the results and the following discussion of the same. I have just a minor comment.
Lines 195-198: in my opinion, this little part could be moved to the Materials and Methods section, because it makes it very easy to understand why you inoculated the milk and for what purpose.

**Response:** Thank you for the suggestion. We moved that sentence to the Materials and Methods section.

6. PLOS authors have the option to publish the peer review history of their article ([what does this mean?](https://journals.plos.org/plosone/s/editorial-and-peer-review-process#loc-peer-review-history)). If published, this will include your full peer review and any attached files.

If you choose “no”, your identity will remain anonymous but your review may still be made public.

**Do you want your identity to be public for this peer review?** For information about this choice, including consent withdrawal, please see our [Privacy Policy](https://www.plos.org/privacy-policy).

Reviewer #1: No

Reviewer #2: No

[NOTE: If reviewer comments were submitted as an attachment file, they will be attached to this email and accessible via the submission site. Please log into your account, locate the manuscript record, and check for the action link "View Attachments". If this link does not appear, there are no attachment files.]

While revising your submission, please upload your figure files to the Preflight Analysis and Conversion Engine (PACE) digital diagnostic tool, <https://pacev2.apexcovantage.com/>. PACE helps ensure that figures meet PLOS requirements. To use PACE, you must first register as a user. Registration is free. Then, login and navigate to the UPLOAD tab, where you will find detailed instructions on how to use the tool. If you encounter any issues or have any questions when using PACE, please email PLOS at [figures@plos.org](mailto:figures@plos.org). Please note that Supporting Information files do not need this step.
